# Human adipose tissue-derived stem cells cultured in xeno-free culture condition enhance c-MYC expression increasing proliferation but bypassing spontaneous cell transformation

**DOI:** 10.1186/s13287-015-0030-4

**Published:** 2015-04-14

**Authors:** Ana CC Paula, Thaís MM Martins, Alessandra Zonari, Soraia PPJ Frade, Patrícia C Angelo, Dawidson A Gomes, Alfredo M Goes

**Affiliations:** Laboratory of Cellular and Molecular Immunology, Department of Biochemistry and Immunology, Institute of Biological Sciences, Universidade Federal de Minas Gerais, Av. Antônio Carlos, 6627, Belo Horizonte, Minas Gerais 31270-910 Brazil; Instituto Hermes Pardini, Av. das Nações, 2448, Vespasiano, Minas Gerais 33200-000 Brazil

## Abstract

**Introduction:**

Human adipose tissue-derived stem cells (hASCs) are attractive cells for therapeutic applications and are currently being evaluated in multiple clinical trials. Prior to their clinical application, hASCs must be expanded *ex vivo* to obtain the required number of cells for transplantation. Fetal bovine serum is the supplement most widely used for cell culture, but it has disadvantages and it is not safe for cell therapy due to the risks of pathogen transmission and immune reaction. Furthermore, the cell expansion poses a risk of accumulating genetic abnormalities that could lead to malignant cell transformation. In this study, our aim was to evaluate the proliferation pattern as well as the resistance to spontaneous transformation of hASCs during expansion in a xeno-free culture condition.

**Methods:**

hASCs were expanded in Dulbecco’s modified Eagle’s medium supplemented with pooled allogeneic human serum or fetal bovine serum to enable a side-by-side comparison. Cell viability and differentiation capacity toward the mesenchymal lineages were assessed, along with immunophenotype. Ki-67 expression and the proliferation kinetics were investigated. The expression of the transcription factors c-FOS and c-MYC was examined with Western blot, and *MYC*, *CDKN2A*, *ERBB2* and *TERT* gene expression was assessed with quantitative PCR. Senescence was evaluated by β-gal staining. Karyotype analysis was performed and tumorigenesis assay *in vivo* was also evaluated.

**Results:**

The hASCs expanded in medium with pooled allogeneic human serum did not show remarkable differences in morphology, viability, differentiation capacity or immunophenotype. The main difference observed was a significantly higher proliferative effect on hASCs cultured in pooled allogeneic human serum. There was no significant difference in C-FOS expression; however, C-MYC protein expression was enhanced in pooled allogeneic human serum cultures compared to fetal bovine serum cultures. No difference was observed in *MYC* and *TERT* mRNA levels. Moreover, the hASCs presented normal karyotype undergoing senescence, and did not form *in vivo* tumors, eliminating the possibility that spontaneous immortalization of hASCs had occurred with pooled allogeneic human serum.

**Conclusions:**

This complete characterization of hASCs cultivated in pooled allogeneic human serum, a suitable xeno-free approach, shows that pooled allogeneic human serum provides a high proliferation rate, which can be attributed for the first time to C-MYC protein expression, and showed cell stability for safe clinical applications in compliance with good manufacturing practice.

## Introduction

Mesenchymal stem cells (MSCs) are fibroblast-like cells with intrinsic characteristics of self-renewal, long-term viability, multilineage differentiation capacity into cells of mesodermal origin (such as osteoblasts, chondrocytes, and adipocytes), and possibly to cells of nonmesodermal origin (the ectodermal [1] and endodermal lineages [2]), hypoimmunogenic, and immunosuppressive properties [3-5]. Studies suggest that MSCs can regenerate tissues by two different mechanisms: (1) the cells can differentiate along a specific lineage pathway, thus replacing the damaged tissue; and (2) through the paracrine release of trophic factors to induce tissue repair by endogenous cells [6]. MSCs can be derived from a variety of adult tissues (for example, bone marrow [7], amniotic fluid [8], adipose tissue [5], dental pulp [9], and so forth). Adipose tissue is a rich and very convenient source of MSCs, usually termed human adipose tissue-derived stem cells (hASCs), which in culture retain markers in common with the other MSCs [10]. The use of hASCs for therapeutic applications has grown substantially in the last years, because the use of stem cells from adult tissues circumvent some ethical issues associated with the application of embryonic stem cells, and because of their accessibility via isolation from lipoaspirates, a disposable byproduct of cosmetic surgery.

Multiple clinical trials are underway to evaluate the use of hASCs in several fields of regenerative medicine [6,11-14]. However, before hASCs can be used in clinical applications, it is necessary to expand these cells *ex vivo* in compliance with current good manufacturing practice (GMP) guidelines to acquire the required number of cells [6,15]. Moreover, quality control assessments must be carried out at all phases of cell manipulation, including functional assays, sterility control [16], and tests to ensure that spontaneous malignant cell transformation has not occurred [6,15,17].

For the successful cultivation of stem cells for therapies, appropriate culture conditions that mimic the physiological conditions *in vivo* and *in situ* are required. hASCs are often expanded in classical culture media, such as minimum essential medium, Dulbecco’s modified Eagle’s medium, RPMI-1640 and DMEM:F12, commonly supplemented with fetal bovine serum (FBS) that serves to provide hormones, proteins, minerals, and several other factors [18]. However, the use of animal-derived components in human cell culture has disadvantages, including the potential for immune reactions [19], the presence of xenogeneic proteins that are internalized or attached on surfaces of cells [20-22], and the possibility of infectious agent transmission [23,24]. Thus, FBS is not a suitable option for patient safety, and novel approaches are being developed to substitute FBS with alternatives such as human AB serum [25-29], thrombin-activated platelet-rich plasma [26,27], platelet-lysate [26,30] and chemically defined medium [31-35].

Concerns that human MSCs may be prone to malignant transformation have been recently raised. In fact, murine bone marrow-derived stem cells have been shown to undergo spontaneous transformation after long-term *in vitro* culture [36]. On the contrary, Bernardo and colleagues [37] showed that human bone marrow-derived stem cells do not display an aptitude for spontaneous transformation. Murine MSCs may be particularly predisposed to spontaneous transformation because there are many differences with regard to mechanisms of transformation in human and mouse cells [38,39]. It is likely that this is due to different genetic/epigenetic control of the genome in the two species. These controversies about the stability of human MSCs highlight the necessity to investigate carefully the stability of human MSCs in long-term cultures before clinical use.

Developing the ideal culture medium remains an important challenge. Recently our group substituted FBS with pooled allogeneic human serum (aHS) to supplement the basal medium for human MSCs cultures [40,41] which provided suitable conditions to expand these cells *in vitro* with an increased proliferation pattern. Thus, our aim in this study was to evaluate the proliferation pattern as well as the resistance to spontaneous transformation of these hASCs during *in vitro* expansion in this proposed xeno-free culture condition. Through this complete characterization of hASCs cultured in medium supplemented with aHS, we observed that aHS provides a high proliferation rate compared with the FBS supplementation, which was attributed for the first time to higher c-MYC protein expression in aHS cultures. Additionally it was shown that hASCs maintain their phenotypic, functional and genetic stability without any evidence of cell transformation.

## Materials and methods

### Pooled allogeneic human serum

aHS was obtained from the whole blood of distinct blood-group-typed donors, as previously described by our group [40]. Donors gave written informed consent according to the approval of this study by the Ethics Committee in Research of the Universidade Federal de Minas Gerais (n° ETIC 11668613.7.0000.5149), advisory board of National Health Council (CNS). No clinical or laboratory evidence of metabolic diseases, hepatitis, HIV, or other systemic complications was observed for the donors. Briefly, the blood was collected from each donor using vacutainer tubes (Biosciences, USA) and allowed to clot spontaneously at 4°C. The serum was separated by centrifugation at 252 *g* at 4°C for 15 minutes. Different blood types (A, B, AB and O) from 20 (five of each blood type) different donors were mixed to produce the batches of aHS. The aHS was inactivated at 56°C for 30 minutes prior to use and kept frozen at −20°C. The batches of aHS were used in the culture of all hASCs studied in this work.

### Culture media

Basal medium consisted of Dulbecco’s modified Eagle’s medium-high glucose (Sigma-Aldrich, USA) supplemented with 5 mM sodium bicarbonate (Cinética Química Ltda, Brazil), penicillin (100 units/mL; Sigma-Aldrich), streptomycin (0.1 mg/mL; Sigma-Aldrich), amphotericyn B (0.25 μg/mL; Sigma-Aldrich), gentamicin (60 mg/L; Schering-Plough, USA), and 10% of either aHS or FBS (Cripion Biotecnologia Ltda, Brazil). Adipogenic medium consisted of basal medium with 0.5 mM isobutylmethylxanthine (Sigma-Aldrich), 200 μM indomethacin (Sigma-Aldrich), 1 μM dexamethasone (Aché, Brazil), and 10 μM insulin (Eli Lilly and Company, USA). Osteogenic medium consisted of basal medium with 50 μg/mL ascorbate-2-phosphate (Ecibra, Brazil), 10 mM β-glycerophosphate (Sigma-Aldrich), and 0.1 μM dexamethasone (Aché). Chondrogenic medium consisted of basal medium with 1 mM dexamethasone (Aché), 125 μg/mL bovine serum albumin (PAA, Austria), 1 mM pyruvate (Sigma-Aldrich), 200 U/mL insulin (Eli Lilly and Company), 3.25 μg/mL transferrin (Wako, Brazil), 0.01 μg/mL transforming growth factor-β1 (Sigma-Aldrich), 5 mg/mL ascorbate-2-phosphate (Ecibra) and a reduced concentration of serum supplements - 1% aHS or 1% FBS [5].

### Isolation and culture of human adipose tissue-derived stem cells

Human adipose tissue was harvested from healthy patients who had abdominal reduction surgery for aesthetic reasons at Núcleo de Cirurgia Plástica, Belo Horizonte, Minas Gerais, Brazil. No metabolic diseases, HIV, hepatitis, or other systemic complications were reported from these patients. The Núcleo de Cirurgia Plástica supplied the information about ABO blood and Rh group of lipoaspirate donors (data not shown). Lipoaspirate from 10 different donors, ranging in age from 20 to 40 years, were assessed in this study. The patients gave written informed consent according to the approval of this study by the Ethics Committee of the Universidade Federal de Minas Gerais (n° ETIC 11668613.7.0000.5149), advisory board of CNS.

The isolation and culture of hASCs was performed as previously described [5]. Briefly, 15 mL of the raw lipoaspirates were washed with phosphate-buffered saline (PBS) and enzymatically digested with 0.075% collagenase type I (Life Technologies, USA) in PBS at 37°C for 1 hour. Subsequently, the stromal vascular fraction was isolated by centrifugation at 252 *g* for 10 minutes, and the pellet was resuspended in basal medium, plated into polystyrene cell culture flasks (T25; Sarstedt, USA) and incubated at 37°C in a humidified 5% CO_2_ atmosphere (Water Jacketed CO_2_ Incubator; Thermo Scientific, USA). The hASCs were maintained under two distinct culture conditions: basal medium with 10% aHS or 10% FBS. After 12 to 28 hours of incubation, the medium was changed and nonadherent cells were removed.

The hASCs were maintained at subconfluent levels, with three weekly medium changes. When the cells reached 80 to 90% confluence, they were washed with PBS and treated with 0.05% trypsin-EDTA (Invitrogen, Canada) for 3 to 5 minutes to detach from the surface of the culture flask. The resulting suspension was replated in new cell culture flask. The hASCs were expanded this way until they were used in the assays. To perform the assays the cells were harvested with basal medium after the treatment with trypsin-EDTA, centrifuged and counted in a Neubauer chamber (HBG, Germany) to seed the cell number accordingly for each assay.

### *In vitro* cell viability and proliferation

Cell viability and proliferation were assessed at passage 4 by the 3-(4,5-dimethylthiazol-2-yl)-2,5-diphenyltetrazolium bromide (MTT) assay (Invitrogen) as previously described [42]. The hASCs were cultured in 24-wells plate (Sarstedt) with basal medium supplemented with aHS or FBS at 37°C in a humidified 5% CO_2_ atmosphere. At the end of each time point (7, 14, 21, and 28 days) the medium was removed and 170 μL of the MTT solution (5 mg/mL) and 210 μL of new basal medium were added to each well. Two hours later, the formazan crystals were dissolved with 210 μL SDS-10% HCl. After 18 hours, 100 μL solution was transferred to a 96-well plate (Sarstedt), and the optical density (OD) was measured at 595 nm using the Anthos 2010 Microplate Reader Standard (Biochrom, UK) and ADAP Basic software (Anthos Labtec, Austria).

### *In vitro* differentiation potential

hASCs were induced to differentiate toward the adipogenic, osteogenic, and chondrogenic lineages for 21 days [5]. For adipogenic differentiation, 1 × 10^3^ cells/cm^2^ were cultured in a 6-well plate (Techno Plastic Products AG, Switzerland) in adipogenic medium with three weekly medium changes. Oil Red O staining (Thermo Scientific) was performed following the manufacturer’s instructions as an indicator of intracellular lipid accumulation. The cells were washed with PBS and fixed in 10% formalin for 1 hour. The cells were then washed with 60% isopropanol and stained with an Oil-Red O (Thermo Scientific) solution in 60% isopropanol for 5 minutes, rinsed with deionized water, and counterstained with hematoxylin for 1 minute. Osteogenic differentiation was induced by culturing 2.5 × 10^3^ cells/cm^2^ in a 24-well plate in osteogenic medium with three weekly medium changes. Differentiation was then assessed by von Kossa staining as an indicator of extracellular matrix calcification. For von Kossa staining, the cultures were fixed in 70% ethanol, incubated with 5% silver nitrate (Vetec, Brazil) and exposed to ultraviolet light for 1 hour. Cells were rinsed with distilled water and 5% sodium thiosulfate (Cinética Química Ltda) and counterstained with eosin for 40 seconds. Chondrogenic differentiation was induced by culturing hASCs in a three-dimensional pellet. For pellet cultures, 5 × 10^5^ cells were centrifuged at 800 *g* for 5 minutes in a 15-ml polypropylene conical tube (Sarstedt). Pellets were then cultured in chondrogenic medium with media changes once a week. Differentiation was assessed by embedding the pellets in paraffin, sectioning them (5 μm sections), and then performing histological staining with Alcian Blue 8GX (1% in acetic acid, pH 2.5; Sigma-Aldrich) for 30 minutes, which stains proteoglycans and glycosaminoglycans, and counterstaining with hematoxylin for 1 minute.

### Flow cytometry analysis

Immunophenotypic analysis of hASCs was performed at passages 4 and 10. The protocol was adapted from a previously published study [43]. Briefly, 5 × 10^5^ cells were incubated with 0.4 μg of each monoclonal primary antibody for 30 minutes at 4°C. After washing with PBS to remove unconjugated primary antibodies, the cells were incubated with Alexa Fluor 488 goat anti-mouse (Invitrogen) as a secondary antibody for 30 minutes at 4°C. Finally, the cells were washed with PBS and fixed with 200 μL 1% paraformaldehyde. As a control, hASCs were also incubated with only the secondary antibody to assess the background fluorescence. The following antibodies were used: CD90, CD166, CD45, CD19, CD105-fluorescein isothiocyanate (FITC), CD44 (HCAM), CD73-phycoerythrin (all from BD Biosciences, USA), CD54-FITC (ICAM; Caltag Medsystems, UK), CD34 (Santa Cruz Biotechnology, USA), HLA-ABC-FITC and HLA-DR-FITC (Abcam, USA). The hASCs were analyzed with a Guava® easyCyte™ 6-2 L Flow Cytometer (Millipore, USA) using Incyte acquisition software (Millipore). A minimum of 15,000 events were acquired for each experimental group. The data were analyzed with FlowJo 7.5.6 software(Treestar, Inc., USA ).

### Ki-67 expression - indirect immunofluorescence

hASCs at passage 4 and 10 were cultured at a density of 2 × 10^4^ cells/cm^2^ on tissue culture polystyrene coverslips (2.2 cm × 2.2 cm) in basal medium with aHS or FBS for 24 hours. Subsequently, the cells were analyzed for Ki-67 nuclear expression. Briefly, the cells were fixed with 4% paraformaldehyde and rinsed with 0.2% Triton-X for 10 minutes. The cells were blocked for 1 hour at room temperature with a blocking buffer containing 1% bovine serum albumin and 5% goat serum in PBS. After washing with PBS, the samples were incubated for 2 hours with rabbit monoclonal anti-human Ki-67 (1:50 dilution; Abcam) and mouse monoclonal anti-human α-tubulin (1:1,000 dilution; Sigma) primary antibodies. Following a wash with PBS, the samples were incubated with Alexa Fluor 488 goat anti-rabbit and Alexa Fluor 555 goat anti-mouse (Invitrogen) secondary antibodies for 1 hour. The nuclei were stained with 1 mg/mL Hoechst 33258 pentahydrate (Invitrogen) for 20 minutes. The cells were then visualized by confocal microscopy (Zeiss LSM 5 Live, Germany). The cell nuclei expressing Ki-67 were counted in 10 different regions of the coverslips using the Cell counter tool of Image J software (National Institutes of Health, USA) to determine the number of Ki-67-positive cells in relation to total cell number in culture, represented as percentage of Ki-67-positive nuclei in a graph*.*

### Proliferation kinetics

To assess proliferation kinetics, the hASCs were expanded and analyzed at each passage until passage 14. A density of 4 × 10^3^ cells/cm^2^ was seeded initially and cultured in T25 culture flask (Sarstedt). Once the cells reached a subconfluence of 90%, they were counted using a Neubauer chamber (HBG) and passaged. The number of population doublings (PD) was calculated starting at passage 2 because the number of plastic-adherent cells could first be determined at passage 1. At each passage, the cells were re-plated at the initial density of 4 × 10^3^ cells/cm^2^ in T25 culture flask. If they did not reach a minimum confluence of 80% after 15 days, the culture was discontinued. PD was determined using the following formula: PD = [log_10_NH – log_10_NS] ÷ log_10_2, where NS is the cell number at seeding (4 × 10^3^ cells/cm^2^) and NH is the cell number at harvest [20]. To calculate the cumulative number of population doublings (CPD), the PD determined for each passage was then added to the CPD of the previous passage.

In parallel, cell population doubling time (DT) was calculated at the phase of exponential growth using the following formula: DT = (TH – TS) × log_10_2 ÷ log_10_(NH ÷ NS), where TS and NS are the time and cell number at seeding, respectively, and TH and NH are the time and cell number at harvest, respectively [26].

A growth curve of hASCs cultured at passage 4 was also generated. Briefly, 2 × 10^2^ cells/cm^2^ were plated in T25 cell culture flasks and cultured. At days 3, 5, 7, 9, and 11, cells from T25 flasks were harvested and counted with a Neubauer chamber (HBG). The fold expansion from seeding at day 0 was calculated and compared for both supplements.

### Transcription factors expression - immunoblotting

Immunoblotting was performed using standard methods [44]. In brief, for c-FOS protein evaluation, hASCs at passage 4 and passage 10 (data not shown) were used and lysed with a lysis buffer (20 mM HEPES, pH 7.0, 10 mM KCl, 2 mM MgCl_2_, 0.5% NonidetP-40). The homogenate was incubated on ice for 10 minutes and homogenized by vortex. Then, the homogenate was centrifuged at 1,500 *g* for 5 minutes to sediment the nuclei. The supernatant was then centrifuged at 16,100 *g* for 20 minutes, and the resulting supernatant consisted of the non-nuclear fraction. To extract nuclear proteins, the isolated nuclei pellets were resuspended in NETN buffer (150 mM NaCl, 1 mM EDTA, 20 mM Tris–HCl, pH 8.0, 0.5% Nonidet P-40), and nuclear lysates were collected after centrifugation at 16,100 *g* for 20 minutes at 4°C. For c-MYC protein evaluation, the hASCs at passage 4 and 10 were washed three times with PBS and harvested by scraping and lysed with NETN buffer. The homogenate was incubated on ice for 10 minutes and then centrifuged at 16,100 *g* for 20 minutes. The resulting supernatant consisted of the cell lysate. Protease and phosphatase inhibitors (Sigma) were added to all buffers.

The immunoblots were performed as described previously [44]. Protein samples (30 μg) were separated on a 10% gradient SDS-PAGE gel by electrophoresis and transferred onto a polyvinylidene fluoride membrane (Millipore). The membranes were blocked with 5% nonfat milk and incubated with the relevant antibodies: rabbit anti-human c-FOS (Sigma) and mouse anti-human Lamin B1 (Abcam) at 1:2,000, rabbit anti-human c-MYC (Sigma) and rabbit anti-human GAPDH (Santa Cruz, USA) at 1:500, and rabbit or mouse peroxidase-conjugated secondary antibody (Sigma) at 1:5,000. Blots were visualized by enhanced chemiluminescence and quantitatively analyzed using Image J software.

### Real-time quantitative PCR

Quantitative (q)PCR was performed to evaluate the mRNA levels of the v-myc avian myelocytomatosis viral oncogene homolog (*MYC*) gene that encodes the human c-MYC protein, the cyclin-dependent kinase inhibitor 2A (*CDKN2A*) gene, also known as *p16*, and v-erb-b2 avian erythroblastic leukemia viral oncogene homolog 2 (*ERBB2*). The evaluation of the telomerase reverse transcriptase (*TERT*) gene was also performed. The gene expression was assessed in hASCs at passage 4 and 10 (n = 3), using glyceraldehyde-3-phosphate dehydrogenase (*GAPDH*) as the reference gene. Total cellular RNA was extracted using Trizol reagent (Invitrogen), as described by the manufacturer. Total samples were treated with DNase (Promega, USA) according to the usage information, and the synthesis of cDNA was performed with a RevertAidTM H Minus M-MuLV RT kit (Fermentas, Lithuania).

The primers used in the qPCR assays were designed using the Primer3 program, version 0.4.0 [45]. The sequences of the forward and reverse primers used for amplification of *GAPDH* [GenBank: NM_002046.5], *TERT* [GenBank: NM_198253.2], *MYC* [GenBank: NM_002467.4], *CDKN2A* [GenBank: NM_000077.4] and *ERBB2* [GenBank: NM_004448.3] are: 5′-GAAGGTGAAGGTCGGAGTCAAC-3′ (GAPDH-F′), 5′-AAGGGGTCATTGATGGCAAC-3′ (GAPDH-R′); 5′-CGGAAGAGTGTCTGGAGCAA-3′ (TERT-F′), 5′-GGATGAAGCGGAGTCTGGA-3′ (TERT-R′); 5′-AGAGTTTCATCTGCGACCCG-3′ (MYC-F′), 5′-AAGCCGCTCCACATACAGTC-3′ (MYC-R′); 5′ -GCCGATCCAGGTCATGATGA-3′ (CDKN2A-F′), 5′- ACGGGTCGGGTGAGAGTG-3′ (CDKN2A-R′); 5′-. GAACTCACCTACCTGCCCAC-3′(ERBB2-F′), 5′- GACCTGCCTCACTTGGTTGT-3′ (ERBB2-R′).

Each qPCR reaction contained 10 μL SYBR Green PCR Master Mix 2X (Applied Biosystems, UK); 10 ng of cDNA; each forward and reverse primer at the optimized concentrations (0.5 μM of *TERT*, 0.3 μM of *MYC*, 0.2 μM of *CDKN2A,* 0.2 μM of *ERBB2* and 0.5 μM of *GAPDH*) and water up to a final volume of 20 μl. The reaction profile was an initial step of 50°C for 2 minutes and a step of denaturation at 95°C for 10 minutes, followed by 45 cycles of denaturation at 95°C for 15 seconds and combined annealing and extension at 60°C for 60 seconds. A “no template control” was made in all the qPCR reactions for each pair of primers containing all the reagents except cDNA. Each sample was tested in triplicate. Reaction specificity was confirmed with melting curves analysis and agarose gel electrophoresis experiments. Standard curves were generated with series of log dilutions of cDNA to calculate the amplification efficiency.

The qPCR reactions were performed using Applied Biosystems 7500 Fast Real Time PCR System (Applied Biosystems), and the data were processed by the 7500 Software, version 2.3 (Applied Biosystems).

The calculation of gene expression was performed using the 2^-ΔCt^ method, where ΔC_t_ = C_t_ value of target gene - C_t_ value of reference gene [46].

### Replicative senescence

The expression of pH-dependent senescence-associated β-galactosidase (SA-β-gal) activity by hASCs was analyzed at each passage until passage 14 using the SA-β-gal staining kit (Sigma) as described by the manufacturer [47,48] and, for hASCs cultured in aHS, they were also evaluated at passage 15 and 17 for a better analysis. Briefly, 5 × 10^3^ cell/cm^2^ were cultured in a 24-well plate for 24 hours. After this time, the monolayer cultures were washed twice with PBS, the hASCs were fixed for 6 minutes with fixation buffer (2% formaldehyde/0.2% glutaraldehyde) at room temperature, washed three times with PBS and incubated for 4 hours with fresh SA-β-gal stain solution [5-bromo-4-chloro-3-indolyl P3-D-galactoside (X-gal), potassium ferrocyanide, potassium ferricyanide, citric acid/sodium phosphate, NaCl, MgCl_2_] at 37°C without CO_2_. The blue-stained cells were then observed under an optical microscope (Olympus IX70, Japan) and photographed using the Image Pro Plus 7.01 software. The cells were counted using the Cell counter tool of Image J to calculate the percentage of cells expressing β-galactosidase in the culture and the results were plotted on a graph.

### Cytogenetic analysis

The karyotyping of hASCs was carried out at passage 4 and 10. When the cells reached 90% confluence, cell division was halted using Kario Max colcemid solution (Invitrogen). Subsequently, the cells were harvested by treatment with 0.05% trypsin-EDTA, resuspended in hypotonic solution 0.075 M KCl (Merck, Brazil) and then fixed in a 3:1 methanol acetic acid solution (Merck) [49]. After spreading the fixed cells on a glass slide, the cells were subjected to G banding and chromosomal analysis according to the International System for Human Cytogenetic Nomenclature [50].

### *In vivo* tumorigenesis in mice

The tumorigenicity assay was performed using 8-week-old *Mus musculus* nude/nude male mice (n = 3 per group). The procedure was approved by the Ethics Committee in Animal Research from the Universidade Federal de Minas Gerais n° 373/2012 and under local ethical guidelines. MDA-MB-231 (breast cancer) cells and FN052 induced pluripotent stem cells (iPS) were used as a positive control for the assay. The FN052 iPS were kindly provided by the National Laboratory of Embryonic Stem Cells, Brazil. As several authors have reported that MSCs cultivated *in vitro* in medium supplemented with FBS can be expanded over multiple cell doublings without tumor or teratoma formation [51] we decided to eliminate the group of hASCs cultured in FBS for this assay to reduce the number of animals involved in this study in accordance with the ethical standards requirements. The procedure was conducted as described before [52]. Briefly, cells (1 × 10^6^) cultured in basal medium with 10% aHS at passage 4 were washed with PBS and then resuspended in PBS with 50% Matrigel (BD Biosciences). Then, the cells were injected in the dorso-lateral area into the subcutaneous space. The animals were monitored for tumor growth. If a tumor was visible and palpable, the animals were euthanized. The tumor was removed, documented photographically and measured with digital caliper. The tumor was fixed for 60 minutes in 10% formalin, routinely processed, embedded in paraffin, and cut into 6 μm sections. The tumor sections were assessed by histological staining with hematoxylin and eosin.

### Statistical analysis

All experiments were done in triplicate with the samples of the 10 different donors. The graphs and the statistical analyses were performed using Graph Pad Prism 5.0. The values are presented as the mean ± standard error of the mean. For the data from each type of experiment, the most suitable statistical analysis was performed: student’s *t*‐test and analysis of variance followed by Bonferroni’s or Tukey’s Multiple Comparison post-tests. Differences were considered significant at *P* < 0.05.

## Results

### Isolation and culture of human adipose tissue-derived stem cells

**Figure 1:**
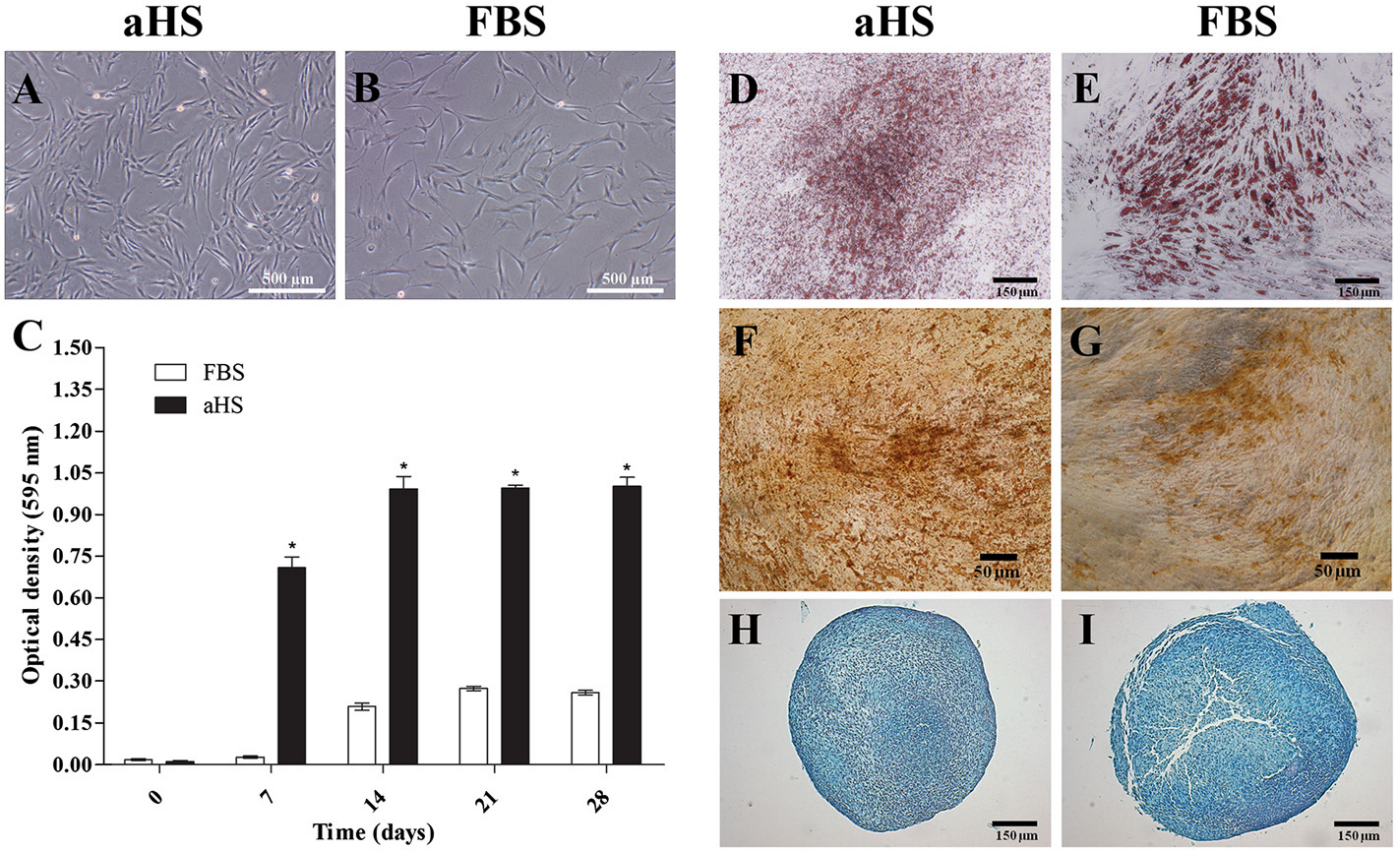
Characteristics of human adipose tissue-derived stem cells from a representative donor at passage 4. **(A,B)** Fibroblast-like morphology of human adipose tissue-derived stem cells (hASCs) cultured in basal medium supplemented with (A) allogeneic human serum (aHS) and (B) fetal bovine serum (FBS); scale bar 500 μm. **(C)** Viability and proliferation of hASCs evaluated by 3-(4,5-dimethylthiazol-2-yl)-2,5-diphenyltetrazolium bromide (MTT) assay performed 7, 14, 21 and 28 days after culture in both culture medium supplements. The results are based on optical density at 595 nm and represent the viability and proliferation of hASCs in basal medium supplemented with aHS and FBS. The results represent the mean ± SEM. Statistical analysis by analysis of variance and Bonferroni*’*s post-test was performed. *P < 0.05, versus FBS . **(D-I)** Multipotentiality of hASCs cultured in basal medium with aHS (left panels) and FBS (right panels). Oil red O staining showed consistent adipogenic differentiation of hASCs cultured in basal medium with (D) aHS and (E) FBS; scale bar 150 μm. von Kossa staining confirmed osteogenic differentiation of hASCs cultured in basal medium with (F) aHS and (G) FBS; scale bar 50 μm. Alcian blue staining verified chondrogenic differentiation of hASCs cultured in basal medium with (H) aHS and (I) FBS; scale bar 150 μm.

The isolation of hASCs using both culture medium supplemented with aHS and FBS was successful. The adherent cells formed several colonies and had a fibroblast-like morphology. However, the hASCs cultured in aHS were smaller and denser than those cultured in FBS (Figure 1A,B). The hASCs also adhered less to the culture plastic surface, as the time required for the cells to detach from the plastic surface during trypsinization was approximately 3 minutes in basal medium with aHS and 5 minutes with FBS. Moreover, when the hASCs cultured in aHS reached confluence they had a tendency to cluster.

### *In vitro* cell viability and proliferation

The hASCs in both types of medium supplements were able to metabolize the MTT into formazan crystals and the OD value enhanced from 7 to 28 days, which demonstrated high viability and proliferation (Figure 1C). When the hASCs were cultured in basal medium with aHS, they exhibited significantly higher OD compared to cultures of hASCs in basal medium with FBS, indicating enhanced proliferation.

### *In vitro* differentiation potential

This assay demonstrated that aHS did not adversely affect the differentiation capacity of hASCs. Intracellular lipid vacuoles were visible by light microscopy inspection of cells under adipogenic induction with aHS (Figure 1D) or FBS (Figure 1E). Osteogenic differentiation was confirmed by the deposition of a mineralized matrix in aHS cultures (Figure 1F) and FBS cultures (Figure 1G). Chondrogenic differentiation was evident in both types of culture conditions, as shown by glycosaminoglycan staining (Figure 1H,I).

### Flow cytometry analysis

**Figure 2:**
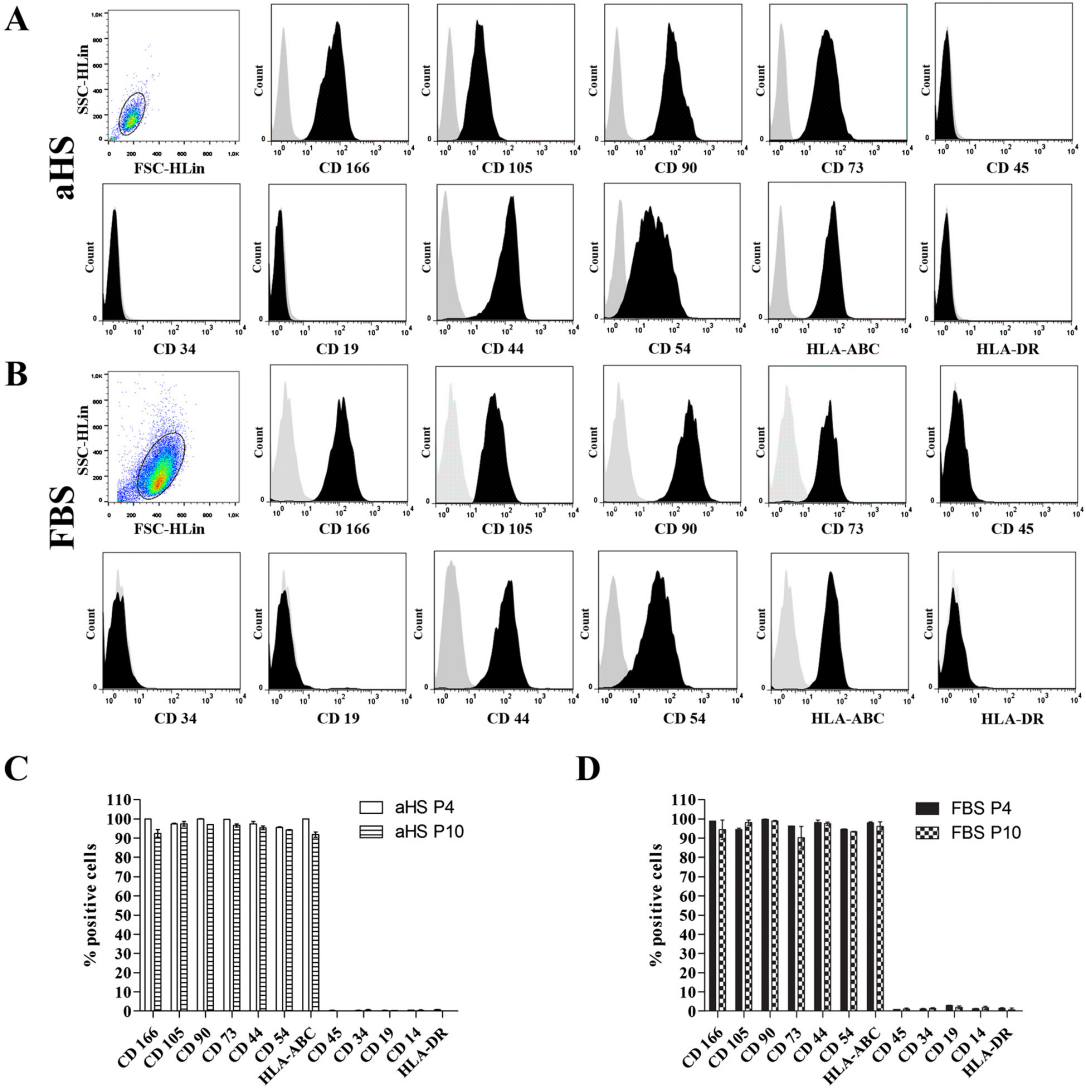
Immunophenotypic analysis of human adipose tissue-derived stem cells. Flow cytometry analysis, where the dot plots represent the cell size and granularity of human adipose tissue-derived stem cells (hASCs) cultured in basal medium with **(A)** allogeneic human serum (aHS) and **(B)** fetal bovine serum (FBS). Expression of the selected mesenchymal stem cell, hematopoietic and human leukocyte antigens markers are depicted with representative histograms for (A) aHS cultures and (B) FBS cultures. The gray peak indicates the isotype-matched monoclonal antibody control. The black peak indicates positively stained cells. The cell populations expressed CD166, CD105, CD90, CD73, CD44, CD54 and HLA-ABC, and did not express CD45, CD34, CD19 and HLA-DR. **(C-D)** Bar graphs represent the quantitative analysis of the hASC expression pattern of markers in basal medium supplemented with (C) aHS and (D) FBS.

Cell size and granularity were measured during the flow cytometry analyses using the forward- and side-scatter settings, and the results showed that the hASCs cultured in basal medium with aHS (Figure 2A) were smaller and had lower granularity compared to cells cultured in basal medium with FBS (Figure 2B). The levels of surface marker expression did not change significantly between the passages or culture medium supplements used. The immunophenotypic profile of hASCs cultured in aHS and FBS indicated that more than 90% of the hASC populations expressed MSC markers CD105, CD166, CD90, CD73 and surface adhesion molecules CD44 and CD54, and the cell cultures lacked the expression of HSC: hematopoietic stem cell; markers CD45, CD34, CD19 and HLA-DR (<2% positive). Moreover HLA-ABC was expressed by approximately 100% of hASCs in both culture conditions and different passages. These results suggest that hASCs cultured in aHS- (Figure 2C) or FBS-supplemented (Figure 2D) medium share a highly similar phenotype.

### Ki-67 expression - indirect immunofluorescence

**Figure 3:**
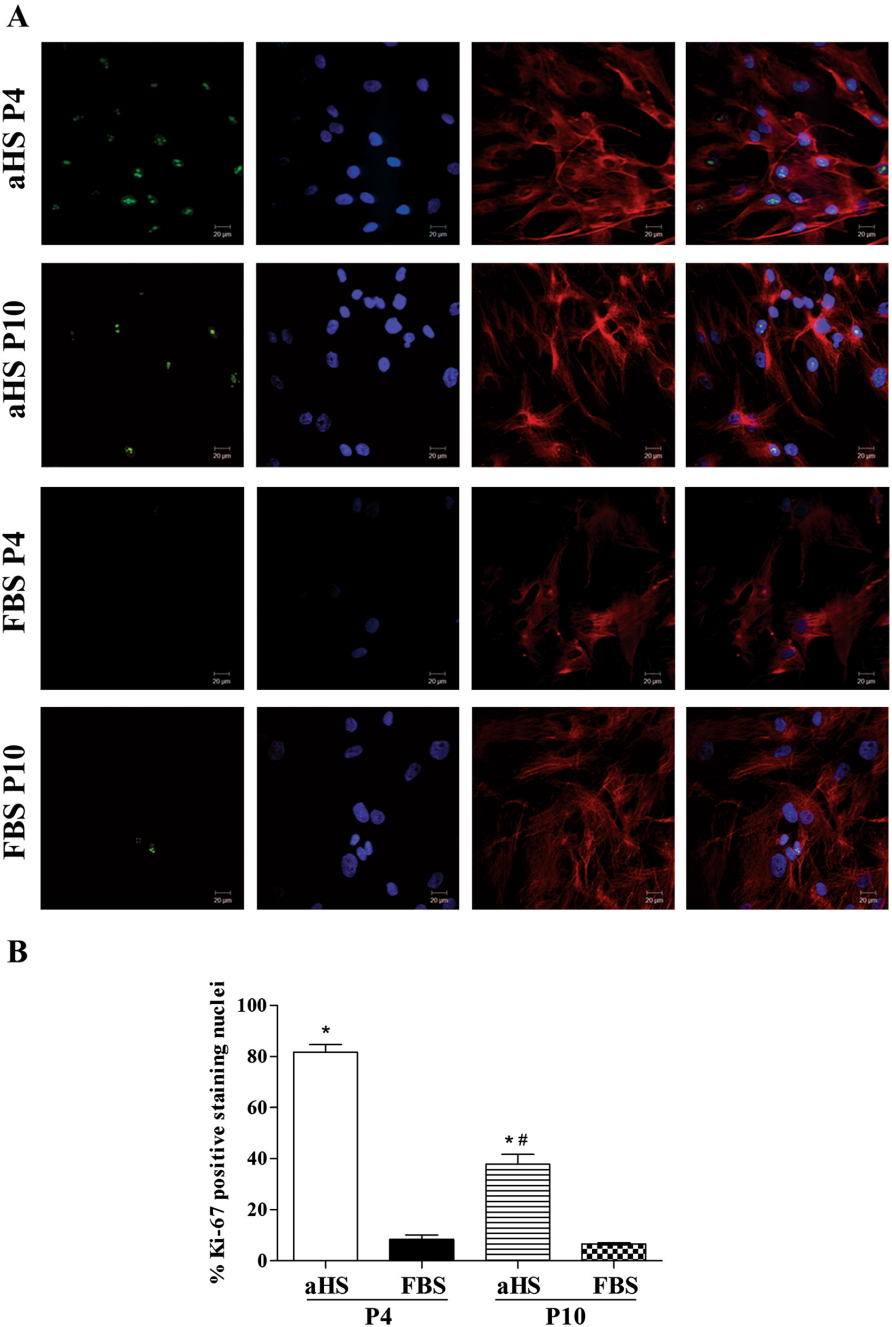
Ki-67 nuclear protein expression in human adipose tissue-derived stem cells. **(A)** Confocal images of the expression of Ki-67 (green) in nuclei and α-tubulin (red), showing the cell shape of human adipose tissue-derived stem cells (hASCs) cultivated in basal medium supplemented with allogeneic human serum (aHS) and fetal bovine serum (FBS) at passages 4 (P4) and 10 (P10). Cell nuclei were stained with Hoechst (blue); scale bar 20 μm. **(B)** Percentage of Ki-67-positive cells/total cells expressed as the mean ± SEM. Analysis of variance and Tukey’s multiple comparison test was performed. *P < 0.05, versus FBS; ^#^ *P* < 0.05, versus P4.

The nuclear expression of Ki-67 protein was evidenced by green nuclear fluorescence, and the red fluorescence indicated the shape of the hASCs (α-tubulin expression; Figure 3A). The estimated percentage of Ki-67-positive cells in culture showed that approximately 81.64% of hASCs cultured in basal medium with aHS at passage 4 were positive for Ki-67, and this number significantly decreased to 37.77% at passage 10. Nevertheless, hASCs cultured in basal medium with FBS presented significantly lower expression of Ki-67, with approximately 8.25% Ki-67-positive cells at passage 4 and only 6.54% at passage 10, but no significant difference was observed between the passages (Figure 3B).

### Proliferation kinetics

**Figure 4:**
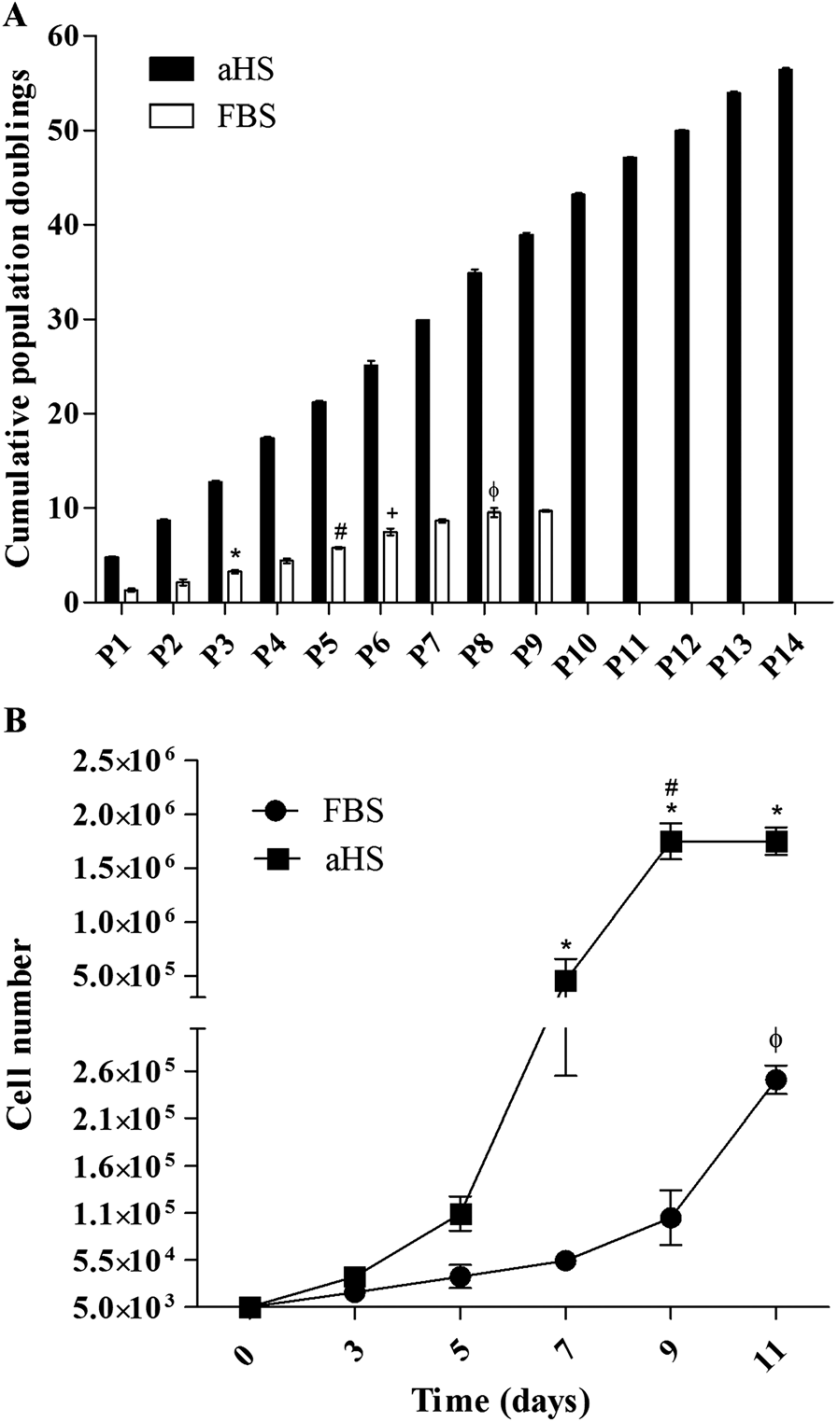
Proliferation kinetics of human adipose tissue-derived stem cells cultured in basal medium supplemented with allogeneic human serum and fetal bovine serum. **(A)** Mean cumulative population doublings measured and expressed as a function of passage number for allogeneic human serum (aHS; black bars) and fetal bovine serum (FBS; white bars). The results represent the mean ± SEM. Analaysis of variance (ANOVA) with Bonferroni's post-test was performed. *P* < 0.05, comparing the condition of basal medium with aHS to basal medium with FBS at all passages and for comparisons between all passages for the condition of basal medium supplemented with aHS. For comparisons between all passages for the condition of basal medium supplemented with FBS: *P < 0.05 for the P1 × P3 comparison; ^#^ *P* < 0.05 for the P3 × P5 comparison; ^+^ *P* < 0.05 for the P5 × P6 comparison; ^ɸ^ *P* < 0.05 for the P6 × P8 comparison. **(B)** Growth curve assessing the proliferation kinetics of human adipose tissue-derived stem cells cultivated in basal medium supplemented with aHS and FBS at days 3, 5, 9, and 11. ANOVA with Bonferroni's post-test was performed. *P < 0.05, comparing hASCs cultured in basal medium with aHS to FBS at days 7, 9 and 11; ^#^ *P* < 0.05, comparing the growth between days 7 and 9 in basal medium supplemented with aHS; ^ɸ^ *P* < 0.05, for the comparison between days 9 and 11 in basal medium supplemented with FBS.

CPD were higher for the cells cultured in basal medium with aHS compared with FBS (Figure 4A). aHS exerted a strong and continuous proliferative effect on hASC growth that was observed up to passage 14. In contrast, hASCs cultivated in FBS exhibited slow but continuous proliferation that was observed only until passage 10. After passage 8, the hASCs cultured in FBS took more than 2 weeks to reach confluence.

For each of the donors, the DT was consistently shorter in hASCs cultured in aHS (24.3 ± 1.9 hours) compared to hASCs in FBS (122.1 ± 1.8 hours).

To better evaluate the period of highest proliferation, growth curves were generated. Notably, the growth curve of hASCs cultured in aHS showed rapid growth starting at day 7, while the accelerated growth of hASCs in FBS did not start until day 9. After day 9, the hASC cultures in aHS had already reached total confluence in the flask and may have stopped proliferating, while the FBS cultures were still proliferating (Figure 4B).

### Transcription factors expression - immunoblotting

**Figure 5:**
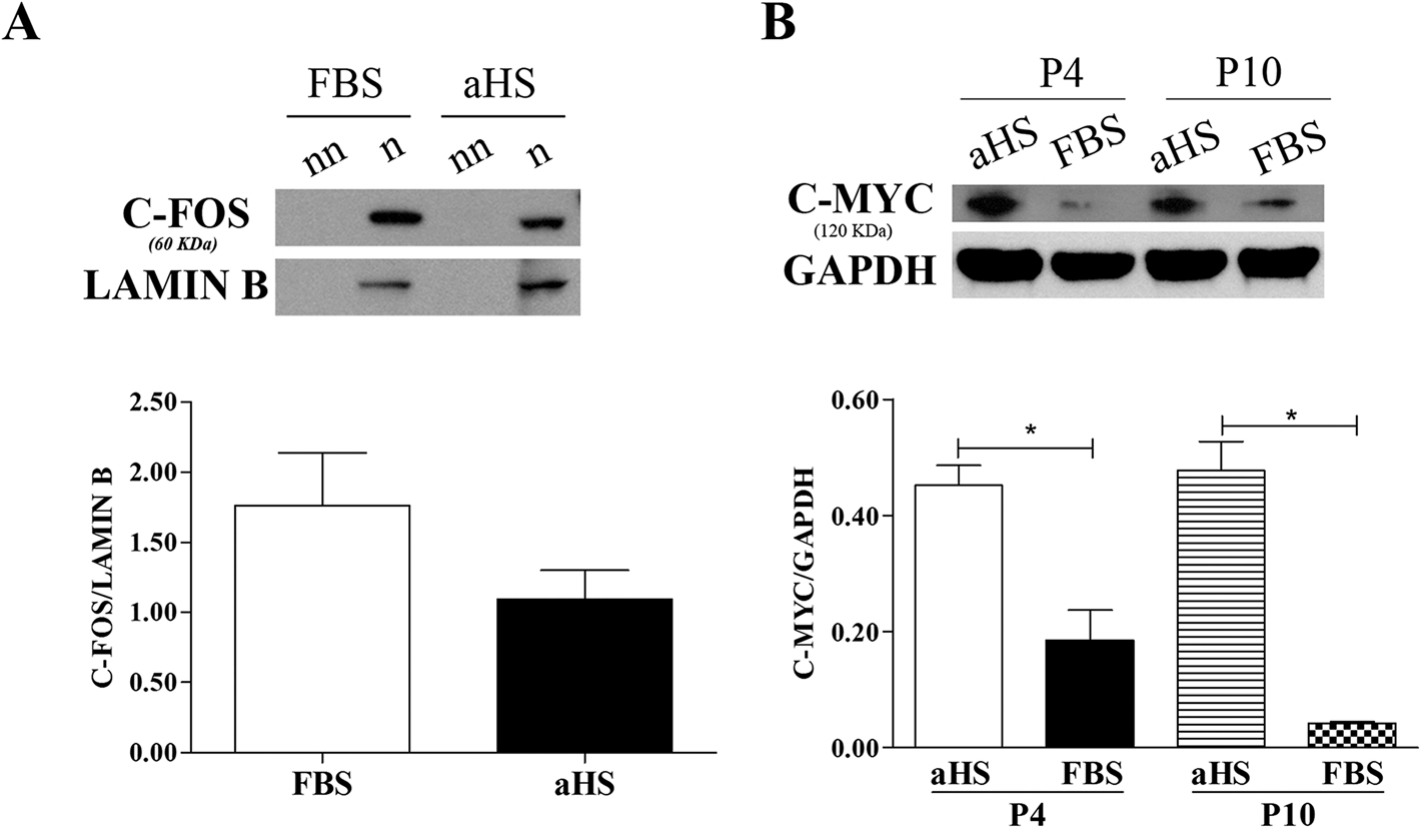
Transcription factors expression of human adipose tissue-derived stem cells cultured in basal medium supplemented with allogeneic human serum and fetal bovine serum. **(A)** Representative immunoblot of c-FOS transcription factor (60 kDa) expression from nuclear fractions of human adipose tissue-derived stem cells (hASCs) at passage 4. Densitometric analyses showed no significant difference in the c-FOS expression in hASCs in both culture medium supplements. Lamin B (60 kDa) was used as a purity control for the nuclear fractions (n, nuclear; nn, non-nuclear). The results were plotted and Student’s *t*‐test was performed. No significant differences were detected (statistical significance was set at *P* < 0.05, two‐tailed). **(B)** Representative immunoblot for c-MYC transcription factor (120 kDa) expression in total extracts of hASCs at passages 4 (P4) and 10 (P10). Densitometric analyses showed that c-MYC in hASCs cultured in basal medium supplemented with allogeneic human serum (aHS) is expressed more than in fetal bovine serum (FBS) in both passages evaluated. GAPDH (37 kDa) was used as a purity control for the total extracts. Bar graph summary showing that hASCs cultured in basal medium with aHS had increased expression of c-MYC compared to cells cultured in basal medium supplemented with FBS. Analysis of variance and Tukey's multiple comparison test was performed. *P < 0.05.

There was no significant difference in c-FOS protein expression level in hASCs cultured in aHS compared to its expression in FBS cultures at passage 4 (Figure 5A) and no significant difference was observed within the groups considering the passages 4 and 10 (data not shown). Nevertheless, a significant difference in c-MYC protein expression level was observed in hASCs cultured in aHS compared to its expression in FBS cultures at both passages (passages 4 and 10), with higher c-MYC expression levels in the hASCs cultivated in aHS. No significant difference was observed within the groups considering the different passages (Figure 5B).

### Real-time quantitative PCR

**Figure 6:**
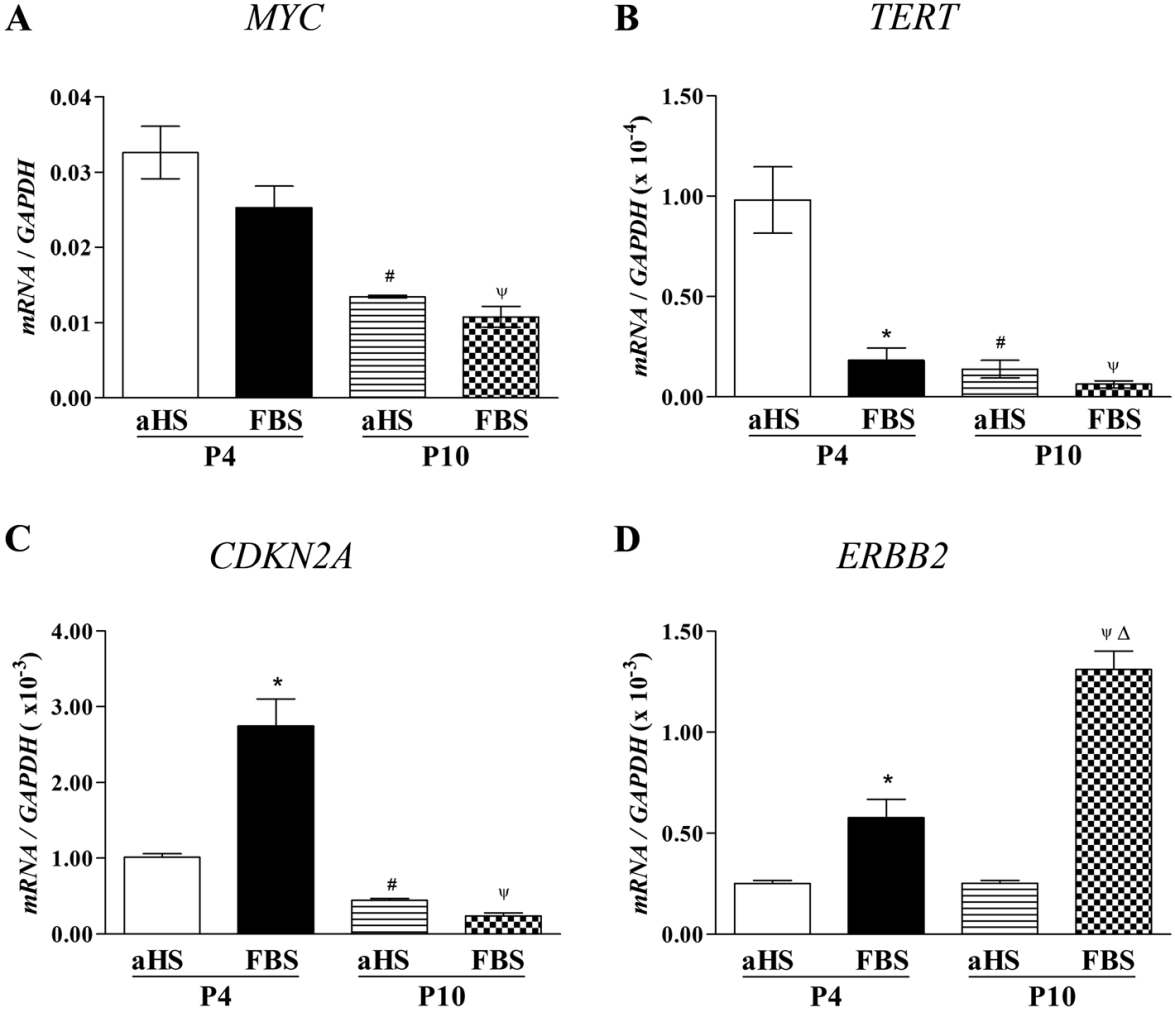
Gene expression of human adipose tissue-derived stem cells cultured in basal medium supplemented with allogeneic human serum and fetal bovine serum. Real-time quantitative PCR analyses showing the expression of **(A)** *MYC*, **(B)** *TERT* **(C)** *CDKN2A* and **(D)** *ERBB2* in hASCs at passages 4 (P4) and 10 (P10). The gene expression was calculated by the 2^-ΔCt^ method and *GAPDH* was used to normalize the data. Analysis of variance and Tukey’s multiple comparison test were performed (n = 3). *P < 0.05, comparing the gene expression of human adipose tissue-derived stem cells (hASCs) cultivated in allogeneic human serum (aHS) versus fetal bovine serum (FBS) at passage 4; ^#^ *P* < 0.05, comparing the gene expression of hASCs cultivated in aHS at passage 4 and passage 10; ^Ψ^ *P* < 0.05, comparing the gene expression of hASCs cultivated in FBS at passage 4 and passage 10; ^Δ^ *P* < 0.05, comparing the gene expression of hASCs cultivated in aHS versus FBS at passage 10.

There was no significant difference in *MYC* mRNA expression level (2^-ΔCt^) in hASCs cultured in aHS at passage 4 (0.0326 ± 0.0060) compared to its expression in FBS cultures at the same passage (0.0253 ± 0.0049). However, *MYC* expression significantly decreased (0.0134 ± 0.0003) in aHS cultures and (0.0108 ± 0.0024) in FBS cultures at passage 10 when compared to passage 4, but no significant difference was observed between the groups at passage 10 (Figure 6A).

Low expression of *TERT* mRNA by hASCs was observed in both basal medium supplements (Figure 6B). There was a significant difference in *TERT* expression levels in hASCs cultured in aHS at passage 4 (0.9803 × 10^−4^ ± 0.2870 × 10^−4^) compared to its expression in FBS cultures at the same passage (0.1824 × 10^−4^ ± 0.10657 × 10^−4^). However, no significant difference was observed comparing aHS cultures (0.1384 × 10^−4^ ± 0.07541 × 10^−4^) with FBS cultures (0.06259 × 10^−4^ ± 0.03165 × 10^−4^) at passage 10. Moreover, there was a significant difference in *TERT* expression comparing passage 4 with passage 10 considering both media (*P* < 0.05).

Low expression of *CDKN2A* mRNA by hASCs was observed in cells cultured in both basal medium supplements (Figure 6C). There was a significant difference in *CDKN2A* mRNA expression level in hASCs cultured in aHS at passage 4 (1.0107 × 10^−3^ ± 0.0479 × 10^−3^) compared to its expression in FBS cultures at the same passage (2.7453 × 10^−3^ ± 0.3577 × 10^−3^). However, no significant difference was observed comparing aHS cultures (0.4415 × 10^−3^ ± 0.0232 × 10^−3^) with FBS cultures (0.2336 × 10^−3^ ± 0.0405 × 10^−3^) at passage 10. Moreover, there was a significant difference in *CDKN2A* expression comparing passage 4 with passage 10 considering both media (aHS and FBS supplements; *P* < 0.05).

Regarding *ERBB2,* low mRNA expression was observed in cells cultured in both basal medium supplements (Figure 6D). There was a significant difference in *ERBB2* mRNA expression levels in hASCs cultured in aHS at passage 4 (0.25000 × 10^−3^ ± 0.01523 × 10^−3^) and FBS cultures at the same passage (0.57743 × 10^−3^ ± 0.09114 × 10^−3^). However, *ERBB2* expression significantly increased (1.31040 × 10^−3^ ± 0.091670 × 10^−3^) in FBS cultures at passage 10 when compared to passage 4, while no significant difference was observed comparing aHS cultures at passage 4 and (0.25168 × 10^−3^ ± 0.013670 × 10^−3^) passage 10. Moreover, there was a significant difference in *ERBB2* expression comparing aHS cultures and FBS cultures at passage 10 (*P* < 0.05**)**.

### Replicative senescence

**Figure 7:**
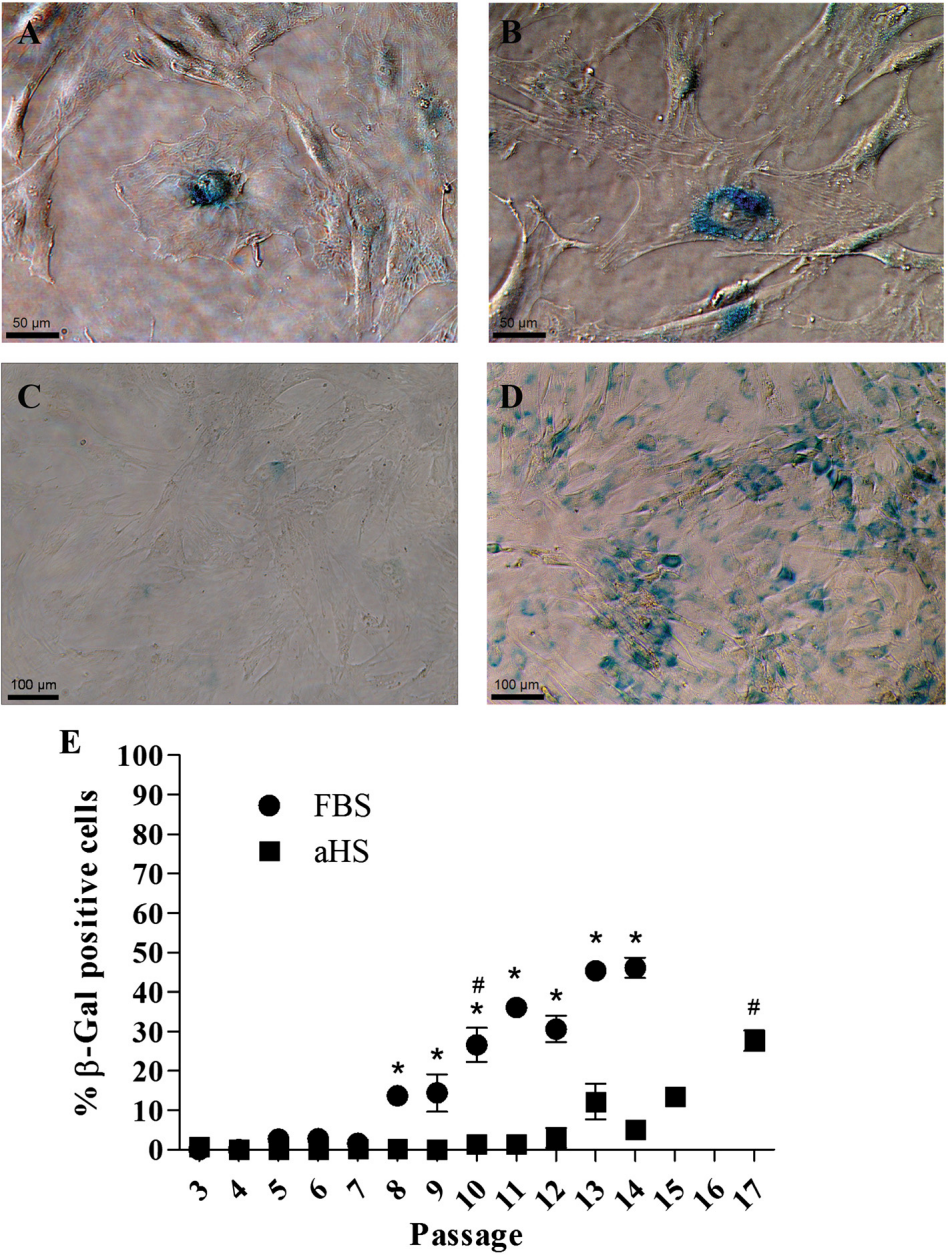
Senescence-associated β-galactosidase expression of human adipose tissue-derived stem cells. **(A-B)** Single cell senescence-associated β-galactosidase (SA-β-gal) staining showing the formation of a blue precipitate in senescent cells (scale bar 50 μm). Representative images of human adipose tissue-derived stem cells **(**hASCs) cultivated in **(C)** allogeneic human serum (aHS) and **(D)** fetal bovine serum (FBS) at passage 10 showing earlier β-gal expression by hASCs cultivated in medium with FBS (scale bar 100 μm). **(E)** Percentage of β-gal-positive cells/total cells at each passage evaluated, expressed as the mean ± SEM. Analysis of variance with Bonferroni’s post-test was performed. *P < 0.05, comparing the condition of basal medium with aHS to basal medium with FBS at each passage after passage 8. ^#^ *P* < 0.05, indicates the passage at which the percentage of senescent cells in the same culture condition were significantly increased.

Senescent cells expressed β-gal that was detected in single cells by X-gal staining. Moreover, the senescent cells presented changes in their morphology due to cell enlargement and the presence of more cytoplasmic vacuoles (Figure 7A,B). Only 1.33% of cells in aHS were positive for β-gal at passage 10 (Figure 7C). However, the hASCs cultivated in FBS expressed β-gal earlier than the cells in aHS, with 25.63% of cells being senescent at P10 (Figure 7D), which is a significant level of senescence compared with the previous passages. The percentage of senescent cells in hASCs cultivated in aHS was significant increased only at passage 17 compared with the previous passages - at this passage, 27% of the cells in culture were senescent. This result indicates a slower senescence process in hASCs cultured in aHS than in FBS (Figure 7E).

### Cytogenetic analysis

**Figure 8:**
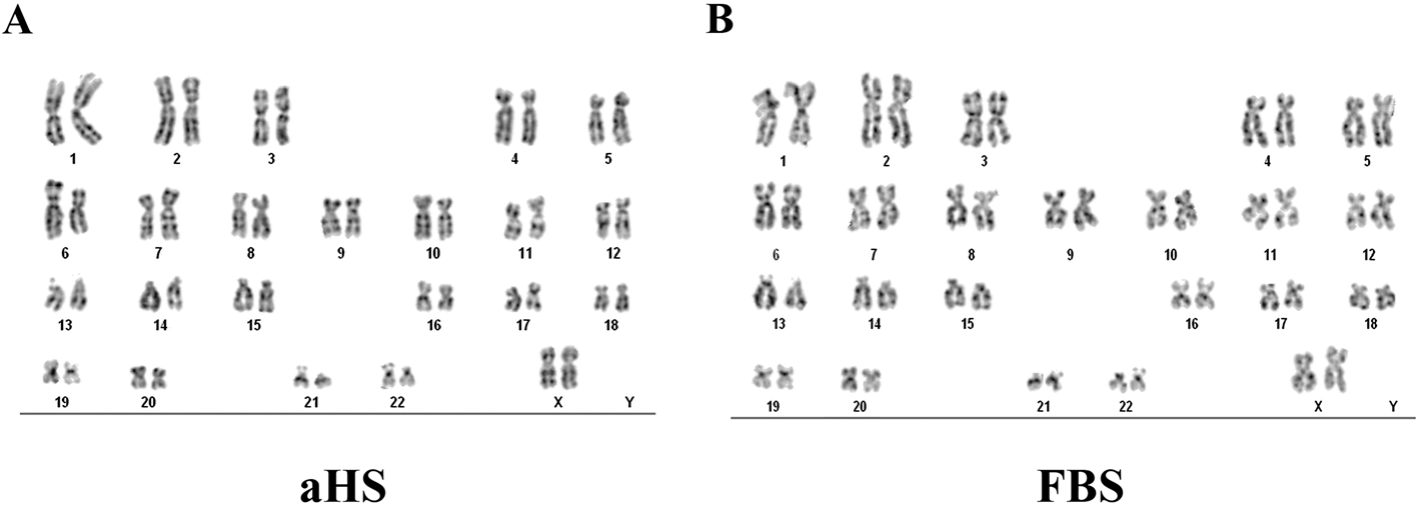
Cytogenetic analysis of human adipose tissue-derived stem cells cultured in basal medium supplemented with allogeneic human serum and fetal bovine serum. Karyotype analysis of human adipose tissue-derived stem cells (hASCs) showed that normal diploid karyotype was maintained after culturing hASCs in basal medium supplemented with **(A)** allogeneic human serum (aHS) and **(B)** fetal bovine serum (FBS), with no abnormal nuclear pattern being observed during long-term culture. The representative karyotypes shown are from hASCs at passage 10.

Karyotype analyses revealed karyotype 46, XX for cells cultivated in both medium supplements, confirming a normal diploid karyotype and demonstrating that no chromosomal alterations had occurred in both passages evaluated (Figure 8).

### *In vivo* tumorigenesis in mice

**Figure 9:**
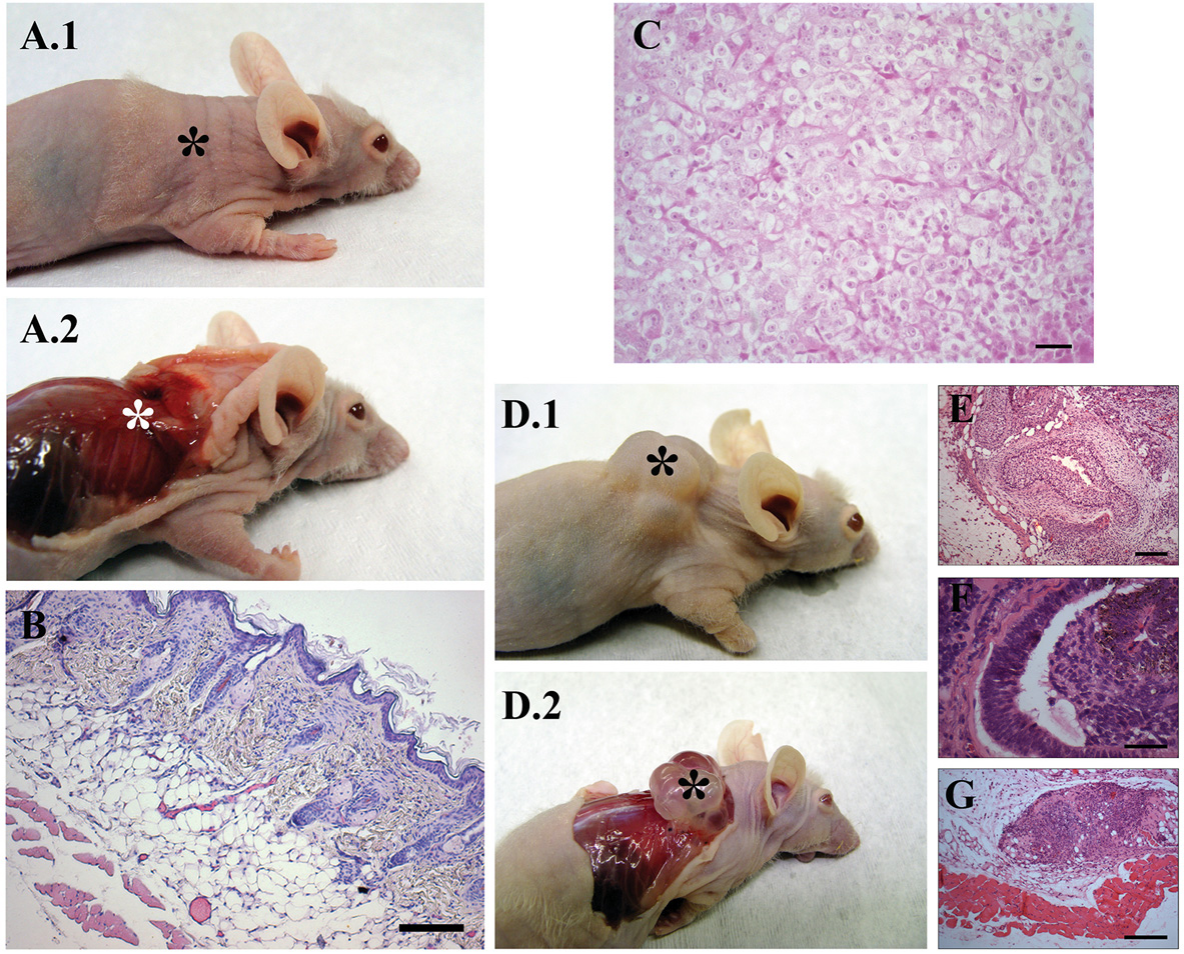
*In vivo* tumorigenesis. **(A)** Human adipose tissue-derived stem cells (hASCs) cultivated in basal medium supplemented with allogeneic human serum (aHS) were injected into the dorso-lateral subcutaneous region of nude mice and did not form tumors. **(B)** Histology of the regions of cell transplantation showed normal skin layers (scale bar 150 μm). (**C**) Histology of tumor formed by transplantation of MDA-MB-231 revealed pleomorphism, mitotic activity and well-delimited tumor (scale bar 30 μm). **(D)** The same numbers of FN052 iPS cells formed teratoma after 12 weeks of transplantation. These tumors involved **(E)** transitional epithelium (endoderm-like tissue; scale bar 150 μm), **(F)** neural ectoderm (ectoderm-like tissue; scale bar 50 μm), and **(G)** skeletal muscle (mesoderm-like tissue; scale bar 150 μm), as shown by histological staining with hematoxylin and eosin. Asterisks indicate the dorso-lateral subcutaneous region of cell transplantation.

The hASCs cultivated in basal medium with aHS were not able to generate tumors in the animals within 24 weeks (Figure 9A). Histological analysis did not reveal any cell or tissue differences other than those expected for subcutaneous tissue, between dermis and muscular tissue (Figure 9B). The animals injected with positive control MDA-MB-231 cells formed malignant tumors after 5 weeks. The tumors presented approximate dimensions of 8.13 mm × 7.02 mm × 5.46 mm. The histological analysis revealed the following features: solid tumor with pleomorphism, mitotic activity and well-delimited tumors (Figure 9C). Moreover, necrosis in the inner area was observed in some tumors. Animals injected with the other positive control FN052 iPS cells formed one clearly identifiable tumor in the locale of the injection after 12 weeks. The tumors presented approximate dimensions of 14.16 mm × 15.86 mm × 8.04 mm (Figure 9D). The tumors were very heterogeneous, and histological analysis revealed tissues from all three germ layers, featuring teratoma. Representative tissue sections stained with hematoxylin and eosin indicated transitional epithelium (endoderm-like tissue; Figure 9E), neural ectoderm (ectoderm-like tissue; Figure 9F) and skeletal muscle (mesoderm-like tissue; Figure 9G).

## Discussion

There are several clinical trials evaluating hASCs [6,11-14]. Current limitations of using MSCs in clinical applications include providing a sufficient number of cells in a timely manner. Furthermore, it is necessary to maintain cell viability and differentiation capacity. There are a considerable number of *ex vivo* MSCs expansion protocols, and FBS has been used to culture diverse cell types for many years.

The serum is important to supplement the cell culture medium because it is a complex mixture of large number of components such as: (1) hormonal factors; (2) transport proteins carrying hormones, minerals and trace elements and lipids; (3) attachment and spreading factors; and (4) stabilizing and detoxifying factors [18]. However, the serum requirements hinder clinical applications. The FBS has several disadvantages: it is an ill-defined medium supplement, and thus an ambiguous factor in MSC culture due to by batch-to-batch variability. Moreover, FBS contains xenogeneic proteins that are internalized or attached on the surfaces of cells [20-22], and can generate immune responses in some patients [53]. Also, it can transmit viruses, prions, bacterias, mycoplasma and endotoxins [23,24].

Therefore, the substitution of FBS with a supplement free of xenogeneic proteins is essential to ensure cell manipulation in accordance with GMP guidelines. Developing an efficient, serum-free and chemically defined cell culture condition is an important issue. Different studies have attempted to replace FBS with human-derived alternatives [25-30,54-56] or serum-free defined media [31-35,57,58].

The serum-free defined media are comprised of synthetic supplements that replace serum and reduce variability, which offer the most promising alternative. Studies show that MSC culture in serum-free media is feasible. However, the MSCs seem to fail to maintain a similar pattern of expansion observed in serum supplemented cultures [57,58].

To date, there is much, but some contradictory, information about the influence of the different types of human substitutes on hASC proliferation and differentiation. Autologous human serum *a priori* is the best replacement for FBS, but limited availability hampers the clinical applicability of autologous human serum for large-scale MSC production [28]. Moreover, studies suggest that natural clotted serum has a better effect in stimulating cell proliferation than plasma, which could be due to the release of certain polypeptides and growth factors from the activated platelets during the clotting process [18,59].

Thus, in this study, we proposed the use of aHS produced from all types of blood groups, which could be available in large amounts for manufacturing and could easily be controlled for quality according to blood banking standards to meet GMP regulations. Additionally, to reduce the variability imposed by different compositions between the serum from different donors, each batch of aHS was constituted of serum from at least 20 different donors (five of each blood type: A, B, AB and O). The choice to use aHS is justified because it has previously been shown that the ABO antigens, as well as other protein and carbohydrate-based antigens, are not detectable on the surface of MSCs, and these cells do not adsorb immunogenic ABO antigens from culture media containing human substitutes [60,61].

The isolations and expansion of hASCs using medium with aHS were successful, and their morphologic characteristics were comparable to hASCs expanded in FBS. The hASCs were fibroblast-like cells, adherent and formed several colonies. The smaller size, reduced granularity, decreased adherence, much denser growth and tendency to cluster at confluence observed for the hASCs cultured in aHS were previously described in a study by our group [40]. These observations also agree with another published study [27] that showed similar characteristics for hASCs cultured in human AB serum and PRP: platelet-rich plasma. Furthermore, whole-genome analysis of hASCs cultured in human AB serum detected lower expression of adhesion and extracellular matrix-associated molecules, supporting the reduced cell adhesion to plastic surfaces during culture with these supplements [62]. However, in this study, the evaluation of the expression of surface adhesion molecules CD44 and CD54 showed that the hASCs did not present lower expression of these proteins when they are cultured in aHS. The cells cultured in aHS expressed approximately 95.50% of CD54 and 98.40% of CD44, while the cells cultured in FBS expressed approximately 94.56% of CD54 and 97.40% of CD44.

The metabolic activity-based assay (MTT) was performed at various time points to determine the cell viability and proliferation when cultured in both types of culture medium supplements, aHS and FBS. MTT results are directly proportional to the number of living cells and they revealed that hASCs cultured in medium with aHS were viable and had a high degree of proliferation compared to cells cultivated in FBS. Also, the cells cultured in medium supplemented with aHS differentiated into adipogenic, osteogenic and chondrogenic lineages as well as the hASCs cultured in basal medium supplemented with FBS.

Immunophenotypic characterization confirmed the smaller size and lower granularity of hASCs cultivated in aHS compared to FBS. Moreover, no significant difference in surface marker expression were revealed between aHS and FBS culture conditions and between the passages evaluated. The majority of hASCs cultivated in both culture supplements were positive for MSC markers (>90%) and had low expression of HSC markers (<2%), which confirms results reported for hASCs cultured with other human supplements [27,28,40,62].

Thus, hASCs cultured in aHS as in FBS were: (1) plastic adherent; (2) viable; (3) expressed MSC-specific surface antigens and lacked expression of HSC markers; and (4) were able to differentiate in multiple lineages under standard *in vitro* differentiating conditions. This is in accordance with the minimal criteria for defining MSCs [3] and hASCs [10] stated and accepted by the International Federation for Adipose Therapeutics and Science and the International Society for Cellular Therapy.

Additionally, because hASCs cultured in basal medium with aHS were denser than with FBS, we evaluated Ki-67 cell expression. This marker is excellent for determining the proliferating fraction of a given cell population because Ki-67 is present in normal cells during all active phases of the cell cycle (G1, S, G2, and mitosis) but it is absent from resting cells (G0) [63]. The results from this analysis confirmed the visual observations of cultures in aHS, as a significantly higher fraction of Ki-67-positive hASCs were found in aHS cultures compared to FBS cultures at passage 4. However, Ki-67-positive cell fractions in aHS cultures decreased at P10 but still remained higher than in FBS cultures.

While this assessment of the proliferating fraction provided information about the state of the cells, it did not provide any information about the rate of proliferation. Therefore, we performed a proliferation kinetics study. The CPD of hASCs was significantly higher for aHS cultures compared to FBS cultures, and CPD trend remained constant throughout passaging. The CPD of cells in basal medium with FBS were assessed until passage 10 because, after passage 8, the cells took more than 15 days to reach confluence. Most likely, this reduced proliferation occurred because hASCs cultured in FBS undergo senescence starting around passage 8, as we have shown and will discuss later. Growth curve analysis allowed us to observe that cells in aHS proliferate faster than cells in FBS at early passages. Moreover, the DT of hASCs cultured in basal medium with aHS was approximately five times shorter than the cells in basal medium with FBS. This proliferation kinetic observed for aHS cultures is clinically relevant and makes aHS an attractive supplement because the large amount of cells required for transplantation could be obtained in a shorter period of time.

Several studies have reported that MSCs cultured *in vitro* can be expanded over multiple cell doublings without loss of differentiation potential. Nevertheless, long-term cultured MSCs can develop chromosomal abnormalities with [64] or without evidence of malignant transformation [51]. Recently, further studies hypothesized that a minimal HT1080 cell lineage contamination could have been present in human MSC cell cultures since the first weeks, and remained undetectable for months until human MSC advanced senescence manifests [65,66] justifying the malignant transformation of human MSCs observed by other studies [64]. Although the majority of human MSC cultures seem to remain stable, the number of expanded cells is linked to the growth rate, and a high proliferative rate may potentiate risk of cytogenetic abnormalities and increase the risks of promoting tumorigenicity in human MSCs, as presented in the report of the conclusions of the meeting with European experts in the field of MSCs [67]. It appears that the majority of cell abnormalities lead to senescence, but it is difficult to exclude the risk of cell transformation. The occurrence of genetic aberrations appears to be related mainly to the manufacturing process rather than to the patient-derived factors [67], but some authors affirm that the susceptibility to malignant transformation can be strictly connected with the origin of the tissue [37,51]. Therefore, it is important to identify and define ideal culture conditions, which avoid the occurrence of chromosomal abnormalities.

In this context, the most important question remains whether this proliferative rate observed in hASCs cultured in basal medium supplemented with aHS is associated with cell transformation. To the best of our knowledge, there is not yet evidence in the current literature that human serum may constitute a suitable and safe replacement for FBS for culture of hASCs. Therefore, this is the first study that investigated the proliferative effects of aHS on hASCs and goes further by carefully evaluating any evidence of spontaneous malignant cell transformation.

Based on the rapid proliferation observed with aHS culture supplementation, we evaluated the expression of the transcriptional factors c-FOS and c-MYC, which are known to transmit proliferative signals and to regulate growth and apoptosis [68,69]. Nevertheless, their dysregulated expression is common in human malignancy [70-72].

The levels of c-FOS expression were not affected in the hASCs cultured in both culture medium supplements. However, the c-MYC expression levels in the hASCs cultivated in aHS were higher than in FBS in both passages evaluated (passage 4 and 10). As a study on murine cells has demonstrated a strong association between overexpression of c-MYC and tumorigenesis [36], this higher expression of c-MYC could lead to spontaneous transformation of hASCs. Nevertheless, the qPCR analysis of *MYC* gene expression revealed similar levels of expression of *MYC* in hASCs cultured in the presence of aHS and FBS at passages 4 and 10. Moreover, *MYC* expression decreased in hASCs cultured in both media at passage 10 compared to passage 4.

Rabbitts and colleagues [73] showed that *MYC* mRNA is present, at equivalent levels, at all times in the cell cycle. Moreover, the *MYC* gene (which is capable of being post-transcriptionally modulated) is activated in the G_0_ to G_1_ by external stimuli (for example, growth factors). An analysis of the c-MYC *in vivo* shows that *de novo* synthesis occurs in G1 and G2, with a protein turnover half-life of approximately 20 to 30 minutes in both phases. Furthermore, the level of c-MYC rapidly increases in the cell population when they re-initiate the cell cycle, thereafter decreasing as the cultures reaches quiescence [73,74]. Thus, the precise mechanism of how this c-MYC protein expression is stimulated by aHS in hASCs remains to be established, but it is possible that c-MYC is a proliferative signal involved in the cell proliferation pattern observed.

Constitutive expression of *TERT*, the gene that controls the length of telomeric ends, has been shown to prevent senescence, increase proliferation and maintain differentiation ability in MSCs [75,76]. Moreover, c-MYC has been described to activate the transcription of *TERT*, which may contribute to sustaining the proliferation of tumor cells along the cell cycle [74]. Therefore, we evaluated the *TERT* expression in hASCs cultured *in vitro*. We detected a low level of *TERT* expression in hASCs cultured in both types of supplement, which helps to eliminate the possibility of spontaneous immortalization of the hASCs in aHS. Our results corroborate other studies showing that MSCs usually have a low level of telomerase activity [26,75].

Prior research has proven that downregulation of the tumor suppressor gene *CDKN2A* (encodes p16 protein) is involved in transformation of MSCs [77,78] and with several cancers [79] and is a valuable marker that assists in the identification of tumorigenic cells [80]. At passage 4, the hASCs cultured in aHS had lower expression of *CDKN2A* compared with FBS cultures. Nevertheless, no significant difference was observed between aHS cultures and FBS cultures at passage 10.

We also evaluated the proto-oncogene *ERBB2* expression, also called *HER2*, that encodes the human epidermal growth factor receptor 2, which is a transmembrane receptor tyrosine kinase. Its oncogenic activation results in its protein overexpression and subsequent abnormal cell signaling, contributing to cancer progression. The overexpression of *ERBB2* has been reported to be associated with different types of malignant tumors in several studies [81-84]. Fortunately, our results showed that the hASCs cultured in both culture supplements presented a low expression of *ERBB2*.

Replicative senescence is associated with the loss of telomeric DNA in each cell division due to incomplete replication of the telomeric ends [75]. As we discussed earlier, the hASCs had low gene expression of human *TERT*. Thus, it was expected that the cells underwent replicative senescence. Our results showed that the hASCs cultivated in basal medium with FBS could be cultivated until passage 8 without any significant senescence, corroborating a previous study [48] in which senescence was found to occur after a CPD ranging from 6 to 16. In contrast, the cells in basal medium with aHS underwent slower senescence and could be cultured until passage 17 without significant senescence. Thus, we can conclude that the pace of senescence is affected by culture conditions. In both cell cultures positive for SA-β-gal, the cells decreased proliferation after they began to undergo senescence. This result may indicate a purposeful program of cell senescence that is triggered irrespective of the culture supplement to protect the cells from accumulating DNA mutations after cell divisions, which could become malignant cells [48].

Importantly, to exclude cells containing abnormalities in aHS cultures, we performed a karyotype analysis which confirmed that no chromosomal alterations had occurred with the hASCs, as numerical and structurally normal karyotypes were observed. This result indicates that the genomic stability of the cells cultivated in aHS was maintained despite their high proliferation.

Despite their proliferative potential, differentiation capacity, and c-MYC expression, the hASCs cultivated in basal medium with aHS did not form tumors *in vivo*, as expected for MSCs. Malignant tumors and teratomas were observed only in the control groups, MDA-MB-231 and iPS, respectively. Teratoma - non-malignant tumor-like formations containing tissues belonging to all three germ layers known as a *sine qua non* characteristic of pluripotent stem cells- was observed only for the iPS control group [52].

In addition to the risk posed by the use of FBS in the *ex vivo* expansion of hASCs for clinical purposes, spontaneous malignant transformation *in vitro* is another potential risk [17]. Some groups have reported that human and murine MSCs *ex vivo* expanded in medium supplemented with FBS become malignant transformed cells [36,78] exhibiting an increased proliferation rate, altered morphology, c-MYC overexpression and high telomerase activity; thus, the cells can escape from senescence and present cytogenetic abnormalities and form tumors when injected into immunodeficient mice [17,36,78]. On the contrary, our results showed that hASCs cultivated in basal medium with aHS had increased proliferation, probably due the increased c-MYC expression, but this proliferation and c-MYC levels showed no association with the *TERT* expression, leading to normal karyotypes. Despite a later senescence compared to FBS cultures, the hASCs cultured in medium with aHS were not able to bypass senescence; moreover, no tumor was generated by these cells. So the results together excluded the possibility of spontaneous immortalization of hASCs. This complete characterization is highly important considering the xeno-free approach proposed to culture hASCs and to ensure a quality and safety standard acceptable for use in clinical applications.

## Conclusions

In conclusion, the results demonstrate that aHS could be easily produced as an off-the-shelf animal-free supplement for the culture of hASCs intended to be used in therapy. Notably, aHS provides a high proliferation rate, which can be attributed to c-MYC protein overexpression in hASCs in this medium compared with medium supplemented with FBS. This higher proliferation of hASCs allows the required cell number to be reached in a short period of time, maintaining phenotypic, functional and genetic stability of the cells throughout long-term culture. This complete characterization ensures that no malignant transformation of hASCs is induced by aHS, and this culture condition supports a safe and effective transplantation into the patient, which is a step forward towards achieving the best GMP criteria.

## Abbreviations

aHS: allogeneic human serum
CPD: cumulative number of population doublings
DT: doubling time
FBS: fetal bovine serum
FITC: fluorescein isothiocyanate
GMP: good manufacturing practice
hASC: human adipose tissue-derived stem cell
HSC: hematopoitec stem cells
iPS: induced pluripotent stem cells
MSC: mesenchymal stem cell
MTT: 3-(4,5-dimethylthiazol-2-yl)-2,5-diphenyltetrazolium bromide
OD: optical density
PBS: phosphate-buffered saline
PD: population doublings
qPCR: quantitative polymerase chain reaction
SA-β-gal: senescence-associated β-galactosidase
